# Light enhanced bone regeneration in an athymic nude mouse implanted with mesenchymal stem cells embedded in PLGA microspheres

**DOI:** 10.1186/s40824-016-0051-9

**Published:** 2016-02-18

**Authors:** Ji Sun Park, Keun-Hong Park

**Affiliations:** Department of Biomedical Science, College of Life Science, CHA University, 6F, CHA bio-complex, 689 Sampyeong-Dong, Bundang-Gu, Seongnam-Si Republic of Korea

**Keywords:** PLGA microspheres, LED, rMSCs, in vivo transplantation, Biomedical devices

## Abstract

**Background:**

Biodegradable microspheres fabricated from poly (Lactic-co-glycolic acid) (PLGA) have attracted considerable attention in the bone tissue regeneration field. In this study, rabbit mesenchymal stem cells (rMSCs) adherent to PLGA microspheres were implanted into athymic nude mice and irradiated with 647 nm red light to promote bone formation. It was found that irradiating rMSCs with high levels of red light (647 nm) from an LED (light-emitting diode) increased levels of bone specific markers in rMSCs embedded on PLGA microspheres.

**Result:**

These increased expressions were observed by RT-PCR, real time-QPCR, immunohistochemistry (IHC), and von Kossa and Alizarin red S staining. Microsphere matrices coated with rMSCs were injected into athymic nude mice and irradiated with red light for 60 seconds showed significantly greater bone-specific phenotypes after 4 weeks in vivo.

**Conclusion:**

The devised PLGA microsphere matrix containing rMSCs and irradiation with red light at 647 nm process shows promise as a means of coating implantable biomedical devices to improve their biocompatibilities and in vivo performances.

## Background

Tissue engineering has traditionally used non-reactive synthetic and natural matrices for specific tissue regeneration [1–3]. However, the fabrication of suitable biocompatible materials creates new opportunities for tissue regeneration in vivo and in vitro in culture systems that mimic the 3D organizations and functional differentiations of tissues. Many researchers in the tissue engineering field have focused on the roles of stem cells [4–7]. However, although stem cells are capable of differentiating to specific cells and making required tissues, they are problematic in terms of degree of proliferation and multi-lineage differentiation [8]. In order to overcome these shortcomings, many researches have attempted to increase stem cell expansion and differentiation [9, 10].

**Scheme 1:**
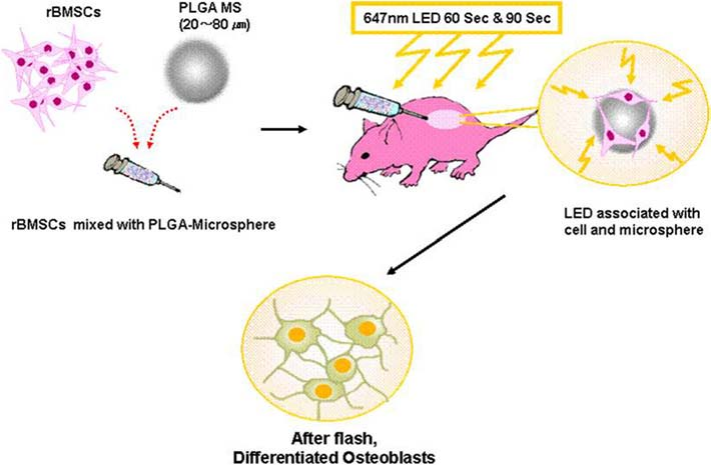
Schematic view of rMSCs embedded onto PLGA microspheres stimulated by LED irradiation

Recently, laser therapy (LT) has been used to stimulate biologic effects in biological systems and cells. Biomodulation induced by light has been the main subject of several reports over the past few years [11–14]. In terms of the application of LT, the wavelength of the light used is thought to specifically stimulate or inhibit actions in cell and tissues. In clinical trials and in vivo, some ranges of wavelength, in particular, red to near IR, were thought be a useful for wound healing [15], peripheral and central nerve regeneration [16], and for the treatment of stomach and duodenal ulcers [17], because such light better penetrates tissues. The several types of cells were found to increase after exposure to low doses of laser irradiation, whereas cell growth and differentiation inhibitory effects were reported at higher doses due to accelerated ATP synthesis in cells [18–23] (Scheme 1).

In the present study, we investigated the use of high doses of laser radiation to increase the differentiation of mesenchymal stem cells (MSCs) embedded in PLGA microspheres for bone regeneration in vivo. In the previous study, we examined the effect of light from a red light-emitting diode on osteogenic differentiation of mouse mesenchymal stem cells (D1 cells) which were cultured in the presence of osteogenic differentiation medium (ODM) for 3 days, then exposed to a red light-emitting diode (LED) light of 647 nm wavelength once for 10 s, 30 s and 90 s with radiation energies of 0.093 J, 0.279 J and 0.836 J, respectively [24]. This study suggested that osteogenic differentiation of mesenchymal stem cells (MSCs) cultured in vitro is enhanced by LED light exposure. Laser irradiation of MSCs offers the potential to promote the productions of bone-specific proteins and extracellular matrix, and ultimately the generation of new bone tissue. In this study, we hypothesized that exposing MSCs embedded in a microsphere matrix to radiation emitted by a light emitting diode in vivo might accelerate the differentiation of MSCs into the osteoblast phenotype and facilitate the synthesis of mechanically functional bone.

## Methods

### Cell harvesting and culture

Rabbit bone marrow stromal cells (rMSCs) were harvested from 3-week-old New Zealand White rabbits, as previously described [25]. In brief, bone marrow (BM) was obtained from the rabbit tibias and femurs via either aspiration or flushing with a 16-gauge needle and a 10-ml syringe containing 1 ml of heparin (3,000 U/ml). After being placed in a 50-ml tube containing 5 ml of low-glucose Dulbecco’s modified Eagle’s medium (DMEM) (GibcoBRL, Grand Island, NY), the BM was centrifuged for 10 minutes at 600 *g* in order to obtain a cell pellet. After the supernatant was removed, the cells were resuspended in 10 ml of low-glucose DMEM containing 10 % fetal bovine serum (FBS) and 1 % antibiotics, and 10^5^ cells/dish were then plated and cultured in 10-cm dishes at 37 °C in a humidified atmosphere of 5 % CO_2_ and 95 % air. Non-adherent cells were removed by changing the culture medium after five days of culture. After two weeks of primary culturing, each dish of cells was passaged into three 10-cm culture dishes at seven-day intervals. rMSCs at passage 3 were used in this study. For the cell seeding and growth test, 100 mg of microspheres and 5 × 10^5^ cells/ml of rMSCs were incubated in a Transwell insert in the culture dish, with gentle shaking. After 2 h of incubation, the unattached cells were removed, and the Transwell inset was incubated for cell growth.

### Preparation of PLGA microspheres

PLGA microspheres (molecular weight 33,000) were fabricated as an oil-in-water emulsion followed by solvent evaporation, as previously described [26]. In brief, PLGA (4 g) was dissolved in 30 mL of dichloromethane. Using a glass syringe and needle (needle gauge; 20G), the polymer solution was dropped into 300 mL of aqueous solution containing 2 w/v% of poly (vinyl alcohol) (PVA) while mixing, using a magnetic stirrer at 600 rpm. The suspension was then gently stirred for 2 to 3 h at 35 °C with a magnetic stirrer at 600 rpm in order to evaporate the dichloromethane, and the microspheres were collected via 2 min of centrifugation at 1500 rpm. The collected microspheres were washed four times in distilled water, and were then lyophilized. The size of the microspheres, as measured by SEM, ranged between 20 ~ 80 μm.

### Scanning electron microscopy (SEM) analysis

Scanning electron microscopy (SEM, Philips 535 M) was used to observe the size and morphology of rMSCs. The morphology was observed after gold coating by using a sputter-coater (HUMMER V, Technics, CA). Argon gas pressure was set at 5 psi, and the current was maintained at 10 mA for 5 min. For observing the morphology of cells attached on the PLGA microspheres, the cells were treated with 2.5 % (v/v) glutaraldehyde in PBS and then fixed in 4 °C overnight. Cells attached on the PLGA microsphere surfaces were rinsed with warm PBS for 5 min and immersed for 1 h in 1 % (w/v) osmium tetroxide dissolved in 0.1 M sodium cacodylate. After being washed twice in deionized distilled water, the samples were dehydrated through a graded ethanol series (25 %, 50 %, 75 %, and 90 %) for 5 min each and were washed three times with 100 % ethanol for 10 min. Ethanol was completely dried by air flow in a clean bench before gold coating.

### Nude mouse implantation and LED irradiation

**Fig. 1:**
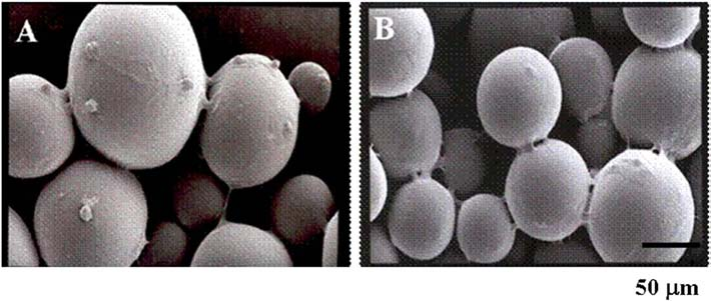
Observation of SEM images of rMSCs adhesion & proliferation on the PLGA microspheres. **a** 3 days & **b** 5 days cultivation

Six-week old Balb/c nude mice were purchased from Clea (Japan). Animal experiments were approved by the Animal Care Committee of CHA University. PLGA microspheres containing rMSCs were transplanted subcutaneously into the backs of mice, which were either exposed or not exposed to red LED light (n = 18/group). Nude mice were anesthetized using 30 μl of 43 % ketamine-7 % rompun. Red light from a 647-nm diode LED generated at 5 mA and 78 mV was used for the irradiation. Anesthetized mice were irradiated for 60 or 90 seconds in a dark room (the 60 and 90 exposured groups; n = 18/group). After being irradiated mice were kept in a dark room until they had recovered from the anesthetic. At 3 weeks after-treatment, mice were sacrificed (n = 6) by anesthetic overdose, and skin areas included transplanted sites were carefully removed for subsequent biological examination. Photographs of the skin flaps were also taken to record the appearance of tissues around treated sites. The method used is illustrated in Fig. 1.

### Reverse transcriptase-PCR (RT-PCR) and real time PCR analysis

Total RNA extraction was conducted using Trizol (Invitrogen, Carlsbad, CA, USA), according to the manufacturer’s instructions. The experiment procedures used were performed as previously described [25]. The oligonucleotides used as primers for RT-PCR and real time-QPCR in this study are described in Tables 1 and 2.

**Table 1:** PCR primer and product size

Gene		Sequence (5′ → 3′)	Size (bp)	Cycle	Annealing temp.	Ref.
COLI	(S)	AGAACATCACCTACCACTGC	250	35	58 °C	Genebank AY633663
	(AS)	ATGTCCAAAGGTGCAATATC				
Cbfa-1	(S)	AGAGGTACCAGATGGGACTGTGGTT	316	35	61 °C	Genebank S83370
	(AS)	GGTAGCTACTTGGGGAGGATTTGTG				
BSP	(S)	CAATAGTGACTCATCCGAAG	280	35	55 °C	Genebank Z46629
	(AS)	CTCCTCATCTTATTCATCAC				
GAPDH	(S)	TCACAATCTTCCAGGAGCGA	293	35	58 °C	Genebank L23961
	(AS)	CACAATGCCGAAGTGGTCGT

**Table 2:** Real time-qPCR primer and product size

Gene		Sequence (5′ → 3′)	Size (bp)	Cycle	Annealing temp.	Ref.
β-Actin	(S)	ACAGAGCCGCCTCTGCC	124	45	58 °C	Genebank AB009345
	(AS)	ACAAGCCTGAGCCGTTGTC				
BSP	(S)	ATACCATCTCACACTAGTTATAATG	116	45	58 °C	Genebank NM000493
	(AS)	AACAGCATAAAAGTGTTCCTATATC				
COLI	(S)	GCAAGAGAGAAAAGAGTGAACC	103	45	58 °C	Genebank AY633663
	(AS)	GTGGCTCAAGCAGAACCAG				
Cbfa-1	(S)	CAGTCACATCAGGATATCC	117	45	58 °C	Genebank L38480
	(AS)	ATGCTGCTGATCTGGAAGA				
OCN	(S)	CTCCAGGCACCCATCTTTAC	121	45	58 °C	Genebank NM00095

### Western blotting analysis

For Western blotting analysis, the cells were lysed in radioimmunoprecipitation assay buffer (RIPA buffer) (Pierce, Rockford, IL, USA) supplemented with a complete protease inhibitor cocktail (Roche Applied Science, Indianapolis, IN, USA). Approximately, 30–50 μg of protein were loaded onto 8-12 % SDS polyacrylamide gels (SDS-PAGE) and then transferred to Immobilon-P membranes (Millipore Corp., Bedford, MA, USA). The membranes were subsequently blocked in 2.5 % skim milk in Tris-buffered saline (TBS)-Tween 20 (0.01 %) and incubated with following primary antibodies: anti-collagen type I (Chemicon, Temecula, CA, USA), anti-BSP (Abcam, Cambridge, UK) and anti-β-actin (Sigma). The blots were visualized by chemiluminescence using Amersham ECL reagents (GE Healthcare, Little Chalfont, UK).

### Histology and immunohistochemical analysis

PLGA microspheres containing rMSCs recovered after 3 weeks in vivo were fixed in 4 % paraformaldehyde, washed in PBS, and incubated for 10 min at room temperature in 1 % Alizarin Red S (Sigma) solution to detect mineralized nodules. For von Kossa staining, fixed and washed cells were incubated in 5 % silver nitrate (Sigma) solution under a 60 W lamp. After 1 h at room temperature, cells were washed in distilled water, and the reaction was stopped by adding 5 % sodium thiosulfate (Sigma) solution for 5 min at room temperature. Finally, cell nuclei were stained by exposure to nuclear fast red (Vector) stain for 3 min. To detect induced proteins, cells were fixed and non-specific epitopes were blocked by incubating them in 2 % BSA for 1 h at room temperature. The cells were then incubated with primary antibodies.

For ALP (Alkaline phosphase) staining, cells were fixed in citrate-acetone-formaldehyde fixative solution for 30 sec at room temperature and then washed with distilled water. Cells were then incubated for 15 min at room temperature and counter stained with hematoxylin.

For immunohistochemical assays, MSCs embedded in PLGA microsphere sections were fixed in 4 % paraformaldehyde solution, dehydrated, and embedded in paraffin, as previously described [25].

### Statistical analyses

The significances of differences between experimental groups were determined using two-tailed Student’s *t*-test. *P*-values of < 0.05 were considered significant.

## Results

### Morphology of rMSCs on PLGA microspheres

Three and Seven days after cultivation, rMSCs adhered onto PLGA microspheres and formed proliferated morphology in vitro (Fig. 1a & b). In 3 days, the rMSCs were observed as adhered on PLGA microspheres, while the rMSCs were proliferated and spread on the whole of the PLGA microspheres.

### RT-PCR analysis

**Fig. 2:**
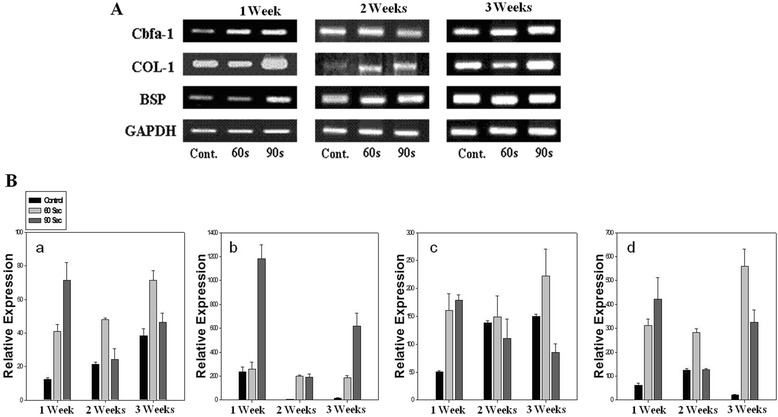
RT-PCR and Quantitative real-time PCR-based analysis of rMSCs embedded in PLGA microspheres stimulated by LED irradiation. (**a**): RT-PCR analysis of mRNA expression of cbfa-1, COL-I, BSP, and GAPDH for 1 week, 2 weeks, and 3 weeks, (**b**): Quantitative real-time PCR-based analysis of mRNA expression of cbfa-1, COL-I, BSP, and OCN for 1 week, 2 weeks, and 3 weeks

RT-PCR was used to examine the expressions of the osteogenic markers collagen type I, BSP, and Cbfa1 mRNAs, which are major bone marker proteins. To determine the effect of LED light on osteogenic differentiation, we transplanted rMSCs mixed with PLGA microspheres because the PLGA microspheres support cell proliferation. Collagen type I was strongly expressed in differentiated cells and its expression was enhanced more in the 90 second exposure groups than in the PLGA control group in 1 week and 3 week transplantation except 2 week transplantation (Fig. 2a). In osteogenic-specific genes of BSP, the expressions stimulated by 60 second were strongly expressed in 2 and 3 week transplantation except 1 week transplantation. With Cbfa1 gene expression, 60 second exposure stimulated the gene expression in whole transplantation.

In order to determine quantitatively expression levels, mRNAs were analyzed by real time-QPCR. Figure 2b showed that the levels of BSP (bone sialoprotein), Cbfa1 (Core binding factor alpha 1), and OCN (osteocalcin) in rMSCs in the 60 second exposure groups increased with time, whereas the PLGA control group showed no specific gene expression (Fig. 2B a, b, c, & d). In particular, the expression of OCN mRNA in rMSCs in the 60 second exposure group was much higher than in the PLGA control group. However, collagen type I gene expression was strongly stimulated by 90 seconds in whole transplantation periods (Fig. 2B b).

### ALP and DAB (3, 3 -diaminobenzidine) staining analysis

**Fig. 3:**
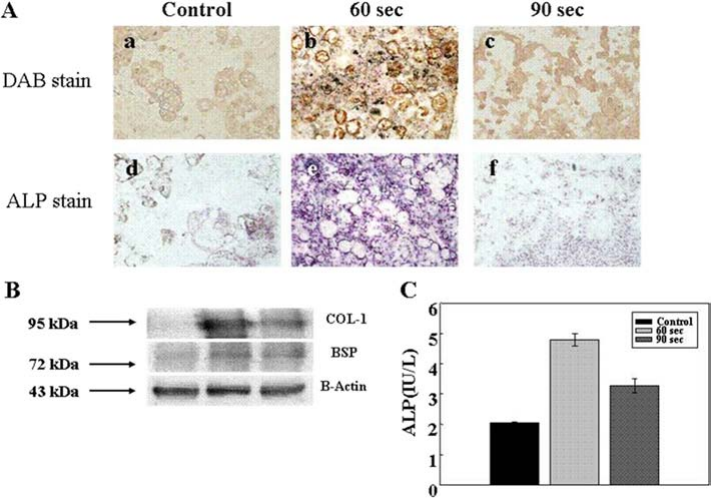
Test for osteogenic differentiation of rMSCs transplanted in nude mouse determined by DAB and ALP staining (**a**), western blotting analysis of β-actin, BSP & collagne type I (**b**), and ALP release from rMSCs stimulated by LED (**c**) in vivo. The bar represents 100 μm

Figure 3a shows ALP and DAB staining findings in the three study groups. The 60 second exposure groups showed higher ALP activities than the PLGA control group after 3 weeks. ALP activity is known to play an important role in the ossification process. Furthermore, ALP activity was significantly higher in 60 second exposure group than in the other two groups; in the 60 second exposure group ALP activity increased with time for two weeks and then leveled off (data not shown).

The specific proteins, collagen type I and BSP, released by differentiated rMSCs irradiated were examined by western blotting (Fig. 3b). Figure 3b shows that collagen type I release from rMSCs onto PLGA microspheres in the 60 second exposure group was highly expressed, whereas BSP (another bone-specific marker) was not. However, the BSP released from rMSCs stimulated for 60 second was more potent than that of other samples.

To evaluate ALP synthesis more precisely, we attempted to quantify its expression as a means of following osteogenic differentiation (Fig. 3c). Our findings suggest, although some rMSCs in the PLGA control group had osteoblastic characteristics, that ALP was expressed at very low levels by cells in the 90 second exposure and PLGA control groups.

### Histologic and immunochemical histologic analysis

**Fig. 4:**
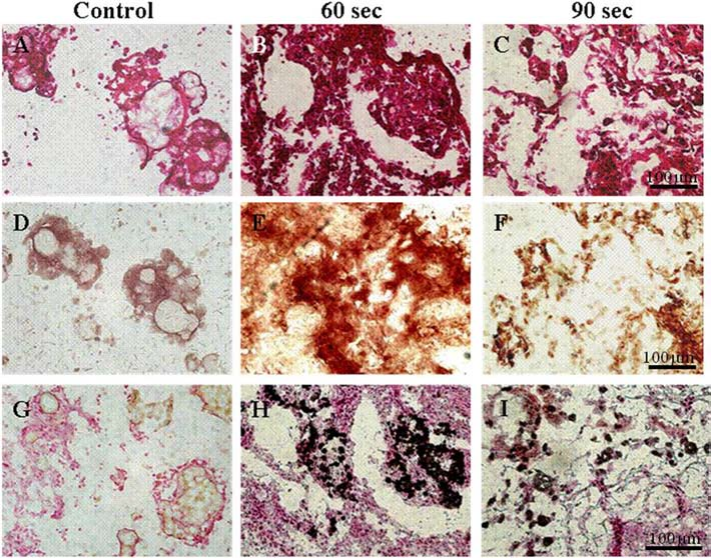
H & E, Alizarin Red S, and von Kossa staining for osteogenic differentiation of rMSCs embedded onto the PLGA microspheres using LED irradiation. (**a**), (**b**), and (**c**): H & E staining, (**d**), (**e**), and (**f**): Alizarin Red S staining, (**g**), (**h**), and (**i**): von Kossa staining

In order to confirm the effect of red light on cell proliferation and differentiation, tissue samples from the two groups were hematoxylin and eosin (H&E), Alizarin Red S, or von Kossa stained. In terms of cell proliferation, H&E staining showed dense cell distributions in the 60 and 90 second exposure groups (Fig. 4a, b, & c). Three weeks after transplantation, samples of the 60 and 90 second exposure groups showed increased osteogenic cell populations (Fig. 4d, e, & f). However, cells in the PLGA control group were wholly undifferentiated (Fig. 4a, d, & g).

In this study, we found that LED red light promoted the mineralization of PLGA microspheres by rMSCs, as evidence by von Kossa and Alizarin red S staining of the calcium deposits released by rMSCs. Furthermore, the amount of calcium released by rMSCs in the 60 second exposure group at 3 weeks post-transplantation (Fig. 4e & h), were substantially greater than in both the 90 second exposure group and the PLGA control group (Fig. 4d & g).

### Immunohistologic assays of collagen type I and BSP

**Fig. 5:**
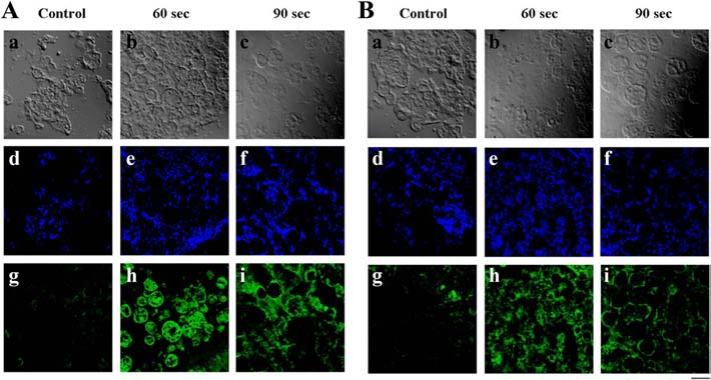
Immunohistochemistry (IHC) staining of collagen type I (**a**) and BSP (**b**) released from rMSCs embedded onto the PLGA microspheres. The bar represents 100 μm

In the 60 second exposure group high levels of collagen type I expression were observed rMSC embedded PLGA microsphere matrices (Fig. 5e & f). However, in PLGA control group no expression of collagen type I was observed. On the other hand, collagen type I expression was smaller in the 90 second exposure group than in the 60 second exposure group (Fig. 5i).

As shown in Fig. 5b, a high levels of BSP expression were also observed when the antibodies to BSP and nuclei was stained with DAPI, in fact, almost all cells in the 60 and 90 second exposure groups stained diffusely positive for BSP (Fig. 5b). However, no BSP was observed in the PLGA control group.

## Discussion

Many studies have been focused on maintaining the differentiation potentialities and expand enough cells for clinical trials. As a suitable source for differentiation, low level light irradiation (LLLI) using 630 nm LED could enhance replicative and colony formation potentials of MSCs derived from human bone marrow [27, 28]. Furthermore, MSCs seeded on three-dimensional (3D) biomatrices were irradiated with LLLI. The consequent phenotype modulation and development of MSCs towards ossified tissue was studied in this combined 3D biomatrix/LLLI system and in a control group, which was similarly grown, but was not treated by LLLI [29].

The osteogenesis indicates that stimulation of light energy to the cells is absorbed by intracellular chromophores [30]. Recent study suggested that low-level laser irradiation generates a small amount of singlet oxygen that influences the formation of adenosine triphosphate (ATP) [31]. Furthermore, laser irradiation may increase the transmembrane electrochemical proton gradient in mitochondria to improve the efficiency of the proton-motive force and generate greater calcium release by an antiport process [32]. A number of different lasers with different wavelengths, including helium-neon (wavelength; 632.8 nm), gallium-aluminum-arsenide (wavelength; 805 ± 25 nm), and gallium-arsenide (wave length; 904 nm), have been used at different intensities and treatment schedules for repairing bone defects. Our findings show that red light treatment of transplanted rMSCs on PLGA microspheres stimulated a cascade of osteogenic events.

## Conclusions

Summarizing, our findings suggest that red light laser LED treatment at 647 nm effectively stimulates osteogenic differentiation in rMSCs embedded in PLGA microspheres. It was found that irradiation for 60 seconds increased the mRNA and protein expressions of bone markers, and increased calcium deposition, and cell proliferation and differentiation in vivo. These results indicate that irradiation with red light has direct and indirect effects on the growth of rMSCs embedded in PLGA microsphere constructs and directs cell differentiation during in vivo tissue regeneration.
